# Methamphetamin abuse a new concern in Iran

**DOI:** 10.1186/2008-2231-20-73

**Published:** 2012-10-31

**Authors:** Omid Mehrpour

**Affiliations:** 1 Birjand Atherosclerosis and Coronary Artery Research Center, Birjand University of Medical Science, Birjand, Iran; 2 Medical Toxicology and Drug Abuse Research Center (MTDRC), Birjand University of Medical Science, Pasdaran Avenue, Birjand, Iran

## Sir

I read with great interest the recent article published by Sadeghi et al., "Report of methamphetamine use and cardiomyopathy in three patients" [1].

The authors state that" cardiomyopathy and acute heart failure may be a new medical concern". Methamphetamine induced cardiomyopathy is certainly a medical concern, and while it is not often reported in Iran, it is a well described clinical finding in the medical literature. Wijetunga et al. retrospectively reviewed 21 crystal methamphetamine hospitalized cases and found that 84% of patients had dilated cardiomyopathy and global ventricular dysfunction in echocardiography. They also concluded that the pathogenesis is probably similar to that of cocaine and catecholamine-induced cardiomyopathy [2].

Moreover, Yeo et al. in a study on 107 cases of cardiomyopathy found that methamphetamine users had a 3.7-fold increased odds ratio for cardiomyopathy, adjusting for age, body mass index, and renal failure. In their retrospective study, 40% of patients under the age of 40 with cardiomyopathy had a history of recent methamphetamine abuse [3].

In addition, as the authors have mentioned, the use of methamphetamine has grown significantly in Iran over the past few years. Complicating the monitoring of drug abuse trends is the street names of illicit drugs are unique to Iran. For exam, Iranian crystal is marketed to Iranian youngsters and the main ingredient is heroin. In central and western Iran, methamphetamine may also be added to this opiate-based drug. Some Iranian addicts (and perhaps researchers) believe that the crystal is actually crystal-meth (amphetamine base) because of the similarity in names [4].

Shishe is another common street drug name in Iran and the main ingredient is methamphetamine.

Iran has the highest rate of opioid drugs addicts in the world [4-6] and with Iranian crystal exposure to methamphetamine may be increasing. Crystal meth and Shishe are also now widely abused.

With the increasing use of methamphetamine, both knowingly (in the cases of crystal-meth and Shishe) and unknowingly (in the case of Iranian Crystal), it is likely that we will see more cases of drug induced cardiomyopathy in the country.
